# Studies of New Fused Benzazepine as Selective Dopamine D3 Receptor Antagonists Using 3D-QSAR, Molecular Docking and Molecular Dynamics

**DOI:** 10.3390/ijms12021196

**Published:** 2011-02-18

**Authors:** Jing Liu, Yan Li, Shuwei Zhang, Zhengtao Xiao, Chunzhi Ai

**Affiliations:** 1 School of Chemical Engineering, Dalian University of Technology, Dalian, 116012, Liaoning, China; E-Mails: lj00772004@mail.dlut.edu.cn (J.L.); zswei@chem.dlut.edu.cn (S.Z.); 2 Center of Bioinformatics, Northwest A&F University, Yangling, Shaanxi, 712100, China; E-Mail: xzt41@126.com; 3 Lab of Pharmaceutical Resource Discovery, Dalian Institute of Chemical Physics, Graduate School of the Chinese Academy of Sciences, Dalian, 116023, Liaoning, China; E-Mail: aicy@dicp.ac.cn

**Keywords:** 3D-QSAR, dopamine D3 receptor, antagonist, CoMFA, CoMSIA

## Abstract

In recent years, great interest has been paid to the development of compounds with high selectivity for central dopamine (DA) D3 receptors, an interesting therapeutic target in the treatment of different neurological disorders. In the present work, based on a dataset of 110 collected benzazepine (BAZ) DA D3 antagonists with diverse kinds of structures, a variety of *in silico* modeling approaches, including comparative molecular field analysis (CoMFA), comparative similarity indices analysis (CoMSIA), homology modeling, molecular docking and molecular dynamics (MD) were carried out to reveal the requisite 3D structural features for activity. Our results show that both the receptor-based (*Q*^2^ = 0.603, *R*^2^_ncv_ = 0.829, *R*^2^_pre_ = 0.690, *SEE* = 0.316, *SEP* = 0.406) and ligand-based 3D-QSAR models (*Q*^2^ = 0.506, *R*^2^_ncv_ =0.838, *R*^2^_pre_ = 0.794, *SEE* = 0.316, *SEP* = 0.296) are reliable with proper predictive capacity. In addition, a combined analysis between the CoMFA, CoMSIA contour maps and MD results with a homology DA receptor model shows that: (1) ring-A, position-2 and R_3_ substituent in ring-D are crucial in the design of antagonists with higher activity; (2) more bulky R_1_ substituents (at position-2 of ring-A) of antagonists may well fit in the binding pocket; (3) hydrophobicity represented by MlogP is important for building satisfactory QSAR models; (4) key amino acids of the binding pocket are CYS101, ILE105, LEU106, VAL151, PHE175, PHE184, PRO254 and ALA251. To our best knowledge, this work is the first report on 3D-QSAR modeling of the new fused BAZs as DA D3 antagonists. These results might provide information for a better understanding of the mechanism of antagonism and thus be helpful in designing new potent DA D3 antagonists.

## 1. Introduction

Being phylogenetically classified as a member of the biogenic amine receptors, DA receptors belong to a large, “rhodopsin-like” subfamily of G-protein-coupled receptors (GPCRs) [1]. In this family, two DA receptor subfamilies named D1- and D2-like receptors exist, the latter of which consists of D2, D3 and D4 subtypes. Both subfamilies couple to GPCRs and inhibit the adenylyl cyclase [1]; however, each subtype mediates different actions of dopamine, such as the dopamine D1 and D2 receptors, which were purported to possess unequal biochemical and pharmacological properties and mediate different physiological functions [1]. Out of all D2-like receptors, more attention is now paid to the dopamine D3 receptors due to their critical role identified in the control of movement [1]. Actually ever since 1990 when the cDNA of DA D3 receptor was first isolated and characterized by Schwartz and co-workers [2], this receptor has attracted much attention due to its antipsychotic activity [3] with a wide body of evidence suggesting its potency in the treatment of schizophrenia and Parkinson’s disease [4]. DA D3 receptor may also be involved in drug dependence and addiction [5]. Hence, due to the diversity of physiological effects, as well as the intimate association with a variety of neuropathological diseases, brain disorders and drug addiction, its specific function and moderate stimulation or depression by antagonists is always quite an active field of scientific and industrial research.

**Figure 1.**
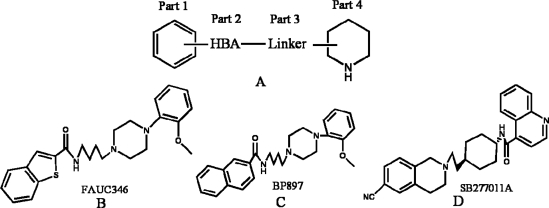
Structures of FAUC346 (**B**), BP897 (**C**) and SB277011A (**D**), with a basic structure of DA D3 receptor antagonists as (**A**) [8–10].

In 2000, a series of DA agonists including quinpirole, quinelo-rane and 7-hydroxy-dipropylaminotetralin developed by Wood *et al.* showed a good correlation between their DA D3 agonist capability and their potency to decrease the cocaine self-administration in rats, suggesting that these agonists mimic or substitute the effects of cocaine [6]. Besides, some selective D3 receptor ligands also reduced the reinforcing efficacy of drugs abuse, and exhibited efficacy in animal models of schizophrenia [7]. The discovery of this possible disease treatment with certain D3 receptor inhibitors has, certainly, aroused another surge of developing preferential D3 partial agonists and antagonists including their analogs [1]. In the field of dopamine D3 receptor antagonists, numerous developments have been observed during the last decade, and possible commonalities in the overall chemical template have been identified among different classes of DA D3 receptor antagonists. Three distinct regions have been typically explored: an aromatic region, a hydrogen bond acceptor region (HBA), and a basic moiety (Figure 1A) [8]. Most of the modifications have been performed on these three regions in order to synthesize novel and more selective D3 antagonists, such as BP897 [6], FAUC346 [9] and SB277011A [10] (Figure 1B–D). However, it is observed that the activity of these derivatives is very sensitive to a slight modification in specific substituents’ positions, which may span from neutral D3 antagonism to modulator activity or partial agonism [8]. Therefore, the exploration of the relationship between the antagonist activity and different structural modifications in the basic structure (Figure 1) of DA D3 receptor ligands is still requisite.

Presently, starting from SB277011A, a series of new fused benzazepine (BAZ) derivatives were synthesized, with 11 diverse kinds of structures including skeleton types A–K (shown in Tables S1–S3, supplementary materials) [7,11]. They attract our research interests not only because they are all DA D3 receptor antagonists, but also due to the fact that their antagonist properties to D3 receptor exhibited a 100-fold selectivity *versus* dopamine D2 and histamine H1 receptors (functional assays) [11]. Thus, it is very promising that they are being developed as new potent selective DA D3/D2 antagonists. In molecular structures, compared with the BP897 and FAUC346 (Figure 1), these new groups of DA D3 receptor antagonists not only possess different Part 4 basic structures but also all have a five-heterocyclic substituent in the aromatic ring (Part 1). To our best knowledge, this series of BAZ is until now the largest dataset (containing 110 compounds) of new fused BAZ-like DA D3 receptor antagonists.

Time consuming and resource costly as the drug discovery and development process is, there is an ever growing effort to apply computational power to the combined chemical and biological space in order to streamline drug discovery, design, development and optimization [12]. Quantitative structure–activity relationships (QSARs), especially the three-dimensional (3D-) QSAR, as one of the computational chemistry areas have been applied widely throughout the world to prioritize untested chemicals for more intensive and costly experimental evaluations [13], which methodologies are also successfully attempted in our previous studies on estrogen receptor subtype binding affinity [14] hepatitis C virus [15], CYP2D6 enzyme inhibitors [16], Catechol-*O*-methyltransferase inhibitors [17] and microRNA-target interaction [18]. The *in silico* studies on DA receptors have also, up to now, achieved some success. For example: DA D3 receptor ligands (FAUC 365 analogues) were studied by using Comparative Molecular Field Analysis (CoMFA) and Comparative Molecular Similarity Indices Analysis (CoMSIA) [4], where only CoMFA and CoMSIA methods were adopted and the whole dataset contained just 47 compounds [4]. To reveal the role of QSAR in DA receptors and antagonist interaction, another group studied 22 individual datasets including DA D(2), D(3) and D(4) receptors, with each dataset containing less than 25 compounds. Finally they found that hydrophobicity is the most important factor in the interactions [19].

The aim of the present study is to use the above mentioned 110 new fused BAZ-like compounds as data set to identify their requisite structural features affecting the dopamine D3 receptor antagonist effects by a combination of several *in silico* approaches. Compared with the above two *in silico* studies, this dataset is not only larger but also contains a different Part 1 and Part 4 basic structure from the former studies. For comparison, both ligand-based and receptor-based QSAR studies, using CoMFA, CoMSIA and molecular docking methods, were carried out. As far as we know, this study provides the first 3D-QSAR study for these new series of DA D3 receptor antagonists.

## 2. Results and Discussion

### 2.1. CoMFA and CoMSIA Statistical Results

**Figure 2.**
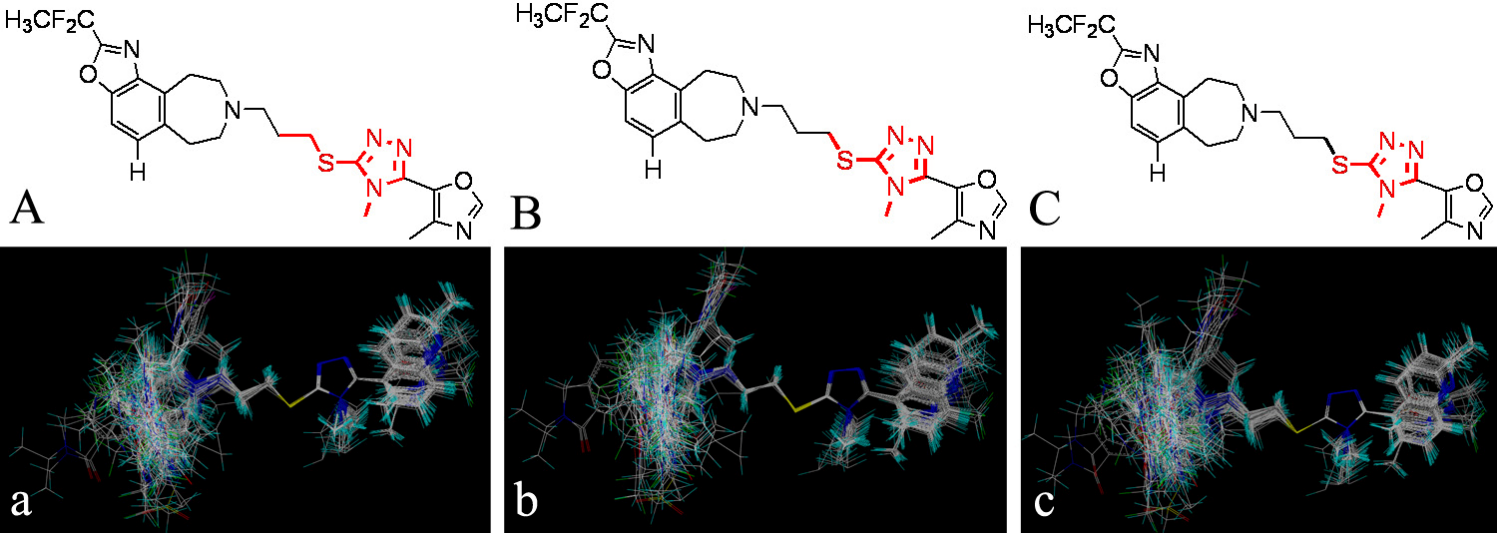
Molecular alignment of compounds in the whole dataset. (**A**), (**B**) and (**C**): three different common substructures of the molecules employed for molecule alignment which are shown in red based on template compound 9; (**a**), (**b**) and (**c**): the ligand-based alignments of all compounds in the dataset based on three different common substructures. Different colors represent different kind of atoms: white for C, blue for N, red for O, green for F, yellow for S and cyan for H, respectively. All the colors in following figures have the same meanings.

It is known that an appropriate superimposition of the molecules being studied within a three-dimensional fixed lattice is the key procedure for further CoMFA and CoMSIA studies [20], thus much effort has been paid to the ligand-based alignment procedure. Firstly, in an attempt to explore the best common substructure for the molecular alignment, we tried three different substructures in building the CoMFA and CoMSIA models (Figure 2), using the same training and test sets employed in all models. During this process, the partial atomic charges of all compounds were calculated by the Gasteiger-Huckel method [21]. Table 1 summarizes the statistical results of the models based on three common structure alignments. Clearly, it is observed that models with common substructure C are best.

Secondly, three alternate charges beside the Gasteiger-Huckel charge [21] (represented as (**A**) in Table 2), *i.e*., the (**B**) Gasteiger-Marsili charge [21], (**C**) Del-Re charge [22] and (**D**) Pullman charge [23], were applied to find the best charge assignments. Table 2 depicts the final results, where the Gasteiger-Huckel charge is demonstrated as obviously the optimal one.

Thus based on the above results, further modeling was carried out using the substructure C and Gasteiger-Huckel charge calculated. Two alignment rules, *i.e*., the ligand- and receptor-based alignments were both employed to overlay the whole 109 compounds, resulting in two different aligned models. All subsequent CoMFA and CoMSIA models were then derived using the same training (82) and test (27 molecules) sets. To determine the reliability of these models, all crucial statistical parameters were analyzed here, including the *Q*^2^ (leave-one-out), *Q*^2^ (leave-group-out), non cross-validated correlation coefficient (*R*^2^_ncv_), *SEE*, F-statistic values and predicted correlation coefficient (*R*^2^_pre_), *R*^2^_boot_ (Bootstrap).

For CoMFA analysis, steric, electrostatic and MlogP were fitted together in every possible form to build appropriate CoMFA models. Finally both the ligand- and receptor-based modeling using descriptors of steric, electrostatic fields and MlogP fields obtained proper reliability (Table 3) and got a result with *Q*^2^ = 0.506, *R*^2^_ncv_ = 0.838, *SEE* = 0.316, *F* = 54.837 with seven optimum components for ligand-based model, and *Q*^2^ = 0.418, *R*^2^_ncv_ = 0.856, *SEE* = 0.292, *F* = 114.875 with four optimum components for receptor-based model, respectively. When being tested by the independent test set, the ligand-based CoMFA model exhibited satisfactory predictive ability with *R*^2^_pre_ = 0.794 and *SEP* = 0.296, but for the receptor-based model the statistical results (*R*^2^_pre_ = 0.481, *SEP* = 0.548) are not good enough. In both CoMFA models, electrostatic feature is found to make more contribution to the activity (∼48% in ligand-based CoMFA and ∼55% in receptor-based CoMFA).

For CoMSIA analysis, five field descriptors concerning with the steric, electrostatic, hydrophobic, HB donor and acceptor interactions were calculated using the same datasets as CoMFA analysis. Combined with MlogP, a total of these six parameters were fitted together in every possible form to build appropriate CoMSIA models. Finally the superior ligand- and receptor-based models were obtained with the highest *Q*^2^ values using steric, electrostatic, hydrophobic, HB acceptor and MlogP parameters (Table 3). The ligand-based CoMSIA model has a *Q*^2^ value of 0.511 with six optimum components, an *R*^2^_ncv_ value of 0.819, a *SEE* value of 0.331 and an *F* value of 56.746. The docking-based CoMSIA model has a *Q*^2^ value of 0.603 with three optimum components, an *R*^2^_ncv_ value of 0.829, a *SEE* value of 0.316 and an F value of 125.886. Furthermore, both CoMSIA models indicate that electrostatic feature plays a major contribution to the antagonist activities.

Normally, 3D-QSAR studies with a *Q*^2^ greater than 0.5 are considered to be statistically significant [24]. In addition, higher *R*^2^_ncv_ and *F* values as well as lower *SEE* values should also be considered as the foundation of a reliable 3D-QSAR model. But the extensively accepted LOO cross-validated *Q*^2^ is insufficient to assess the predictive power of the QSAR models [25]. Here we validate the models by predicting the activity (fpKi value) of the compounds in the test set using the above four models. For this purpose, the test set (27 molecules) which represents 32.9% of the training set, was used here to validate the accuracy of both the ligand- and receptor-based models.

Before the final validation by the test set, an initial inspection of the fitted/predicted activities revealed poor prediction for several inhibitors which were considered as outliers in this work. For the ligand-based models, outliers are compounds 24, 29, 34, 81, 88 and 104; for the receptor-based ones, outliers are 6, 24, 34, 68, 81, 88 and 97. Several reasons, like unmatched structure, different active conformation or more specific molecular mechanisms, may result in the existence of outliers. A particular careful examination of the outliers may provide additional information determining their peculiarities; therefore, in this study all outliers were attentively checked and finally divided into three groups.

(1) Compound 24 has a special substructure of aromatic ring-A which is different from any other chemicals in the data set, and ring-A in our further analysis has been identified as very important in affecting the activity; Molecule 81 is the only antagonist in the dataset with a type *b* R_3_ substituent ( 
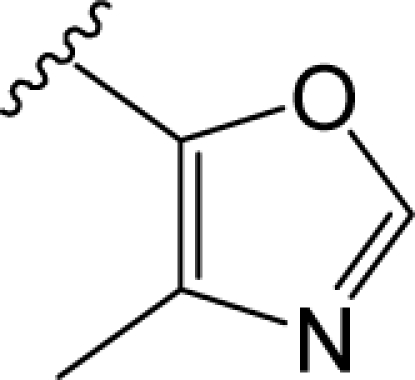
) in skeleton type H group of compounds 76–100. This unique molecular structure may cause the larger standard deviations from the mean of the residuals for the two compounds.

(2) In structure, compound 34 belongs to type E skeleton which includes 33–39, a total of seven BAZ-based derivatives with antagonist activity ranging from 6.6 to 8.1. Though having no unique substructure, compared with others in this group, 34 exhibited an extremely lower (also the lowest in the dataset) fpKi value of 5.6. The case of molecule 29 is similar to 34. Throughout the whole dataset, it is easy to observe that when falling into same skeleton type and possessing same R_1_, R_2_ substituents, all molecules with type a R_3_ substituent ( 
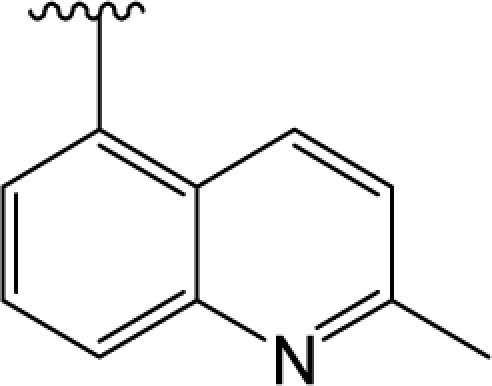
) are always more active than those with *b* type of R_3_ group. However, 29 is an exception, exhibiting lower biological activity than corresponding molecule 30. Whether this disagreement in variance of the structure-activity is due to different binding conformation or specific interaction mechanism still needs further experiments to determine.

(3) As to compounds 6, 68, 88, 97 and 104, they all have a higher residual between the experimental and predicted activity (fpKi residual is larger than 0.7–0.9) and thus are treated as outliers. This discrepancy, we speculate, on the one hand indicates that these particular BAZ derivatives may not be typical of the rest of the data, and on the other hand, suggests the necessity to recruit more plenteous and accurate experimental data with more diversified molecular structures.

After elimination of these outliers, both the CoMFA and CoMSIA models obtained from the ligand-based alignment exhibit good prediction (*Q*^2^ is larger than 0.5 and *R*^2^_pre_ is larger than 0.71) (Table 3), indicating the ligand-based alignment rule is good. For receptor-based alignment, CoMSIA model exhibits good prediction (*Q*^2^ is 0.603 and *R*^2^_pre_ is 0.690), but CoMFA model (*Q*^2^ is 0.418 and *R*^2^_pre_ is only 0.481) is not good enough.

In addition, besides the external test set, another important procedure, *i.e.*, the progressive scrambling was also used to validate the predictive ability of the models with the same principal component numbers as adopted in Table 3, which gauges the dependence of the model on chance correlations [26]. As seen from Table 3, in almost all cases the *Q*^2^_scrambling_ values are slightly lower than the *Q*^2^ values. This is reasonable because *Q*^2^_scrambling_ values are known to be more conservative than those of LOO/CV PLS *Q*^2^ ones [27]. And the calculated cross-validated standard deviations of error of prediction (CSDEP) are slightly larger than the SEP values. By this comparison, receptor-based models show their superiority to the ligand-based ones, due to the former’s much more reasonable *Q*^2^_scrambling_ values for CoMFA and CoMSIA models than the latter’s. Thus, subsequently the optimal model in this study, *i.e.*, the CoMSIA receptor-based model, was utilized for further assessment and discussion.

**Figure 3.**
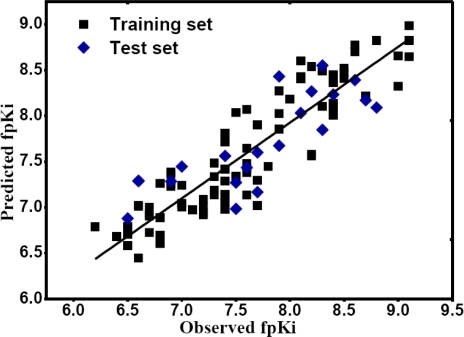
The receptor-based correlation plots of the predicted *versus* the actual fpKi values using the training set (filled black square) and the test set (filled blue diamond) based on CoMSIA model.

The observed and CoMSIA predicted DA D3 receptor inhibitory activities for both receptor-based and ligand-based models are shown in Table S5. Figure 3 depicts the actual *versus* predicted fpKi values plot for both the training (filled black square) and test (filled blue diamond) set molecules of the whole dataset based on receptor-based CoMSIA model. As observed, all the points are rather uniformly distributed around the regression line in this figure and the predicted activities are almost as accurate as the experimental data, indicating a proper correlation between the predicted and experimental activities of the dataset and the reliability of the obtained models.

### 2.2. MlogP Contribution

Hydrophobicity is one of the most crucial properties related to biomolecular interactions, which can be interpreted in terms of the association of non–polar groups or molecules in an aqueous environment which arises from the tendency of water to exclude non–polar molecules [28]. To quantitatively depict the hydrophobicity of a molecule, various molecular descriptors are used, in which lipophilicity represented by logP is most common [29]. Traditionally, this descriptor depicts the lipophilicity of a molecule, which is the logarithm of the partition coefficient of the molecule in a lipidic phase and an aqueous phase. It represents the tendency of the compound to prefer a lipidic environment to an aqueous one. In our present work, MlogP, a calculated logP parameter was calculated by Dragon 5.4 software [30]. The principle of MlogP calculation was invented by Moriguchi *et al.* who carried out a multiple regression analysis of a set of 1230 organic molecules including general aliphatic, aromatic, and heterocyclic compounds, together with complex drugs and agrochemicals when deriving their ‘simple method’ of calculating logP [31]. The larger MlogP value a molecule possesses, the higher lipophilicity and larger hydrophobicity it has. Whereas, hydrophobic field in CoMSIA is another hydrophobic index, describing the specific distribution of the hydrophobic property in 3D-space or surface area of a molecule [32]. In CoMSIA methods, steric, electrostatic, HB donor and acceptor interactions are all determined using a probe atom with a charge of +1, 0 or −1, and the corresponding field points are then placed at the extrema of the interactions [33]. But for the hydrophobic field, the field points are positioned at the centre of hydrophobic groups such as phenyl, halogens and alkyl, and then these field points are used to describe molecules and compare their similarity [33]. Presently, a correlation regression analysis was carried out to MlogP and the hydrophobic field indices in CoMSIA, and the result showed that they were totally independent of each other.

To exploit the impact of hydrophobicity of the molecules on their biological activity (fpKi here), MlogP is used as a descriptor in our PLS analysis in both the CoMFA and CoMSIA analyses. But before this, 3D-QSAR analyses were first performed without MlogP parameter employed using the same training/test set and molecular alignment rule, and Table 4 shows the optimal results. Obviously, it can be seen from the table that without MlogP descriptor, all models’ *Q*^2^ and *R*^2^_pre_ values are much lower and no CoMFA or CoMSIA models with satisfactory statistical results could be obtained, further demonstrating the crucial role MlogP parameter plays in building appropriate 3D-QSAR models. This conclusion is confirmed by the study of Corwin Hansch and his colleagues, in which they studied the role of hydrophobicity (the main parameter is ClogP) of 22 individual series of antagonists (every series of antagonists has less than 21 compounds) binding to DA receptors, and finally found that the hydrophobic term is the most important factor in the DA interactions [19].

### 2.3. 3D-QSAR Contour Maps

**Figure 4.**
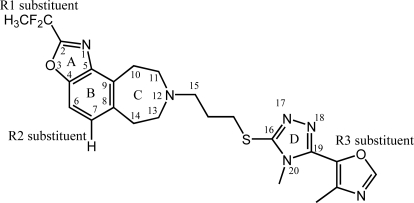
The structure of compound 9 [11]. R1, R2 and R3 substituents are –CF_2_CH_3_, H and 
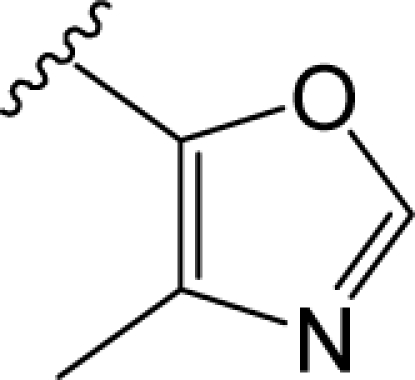
, respectively.

**Figure 5.**
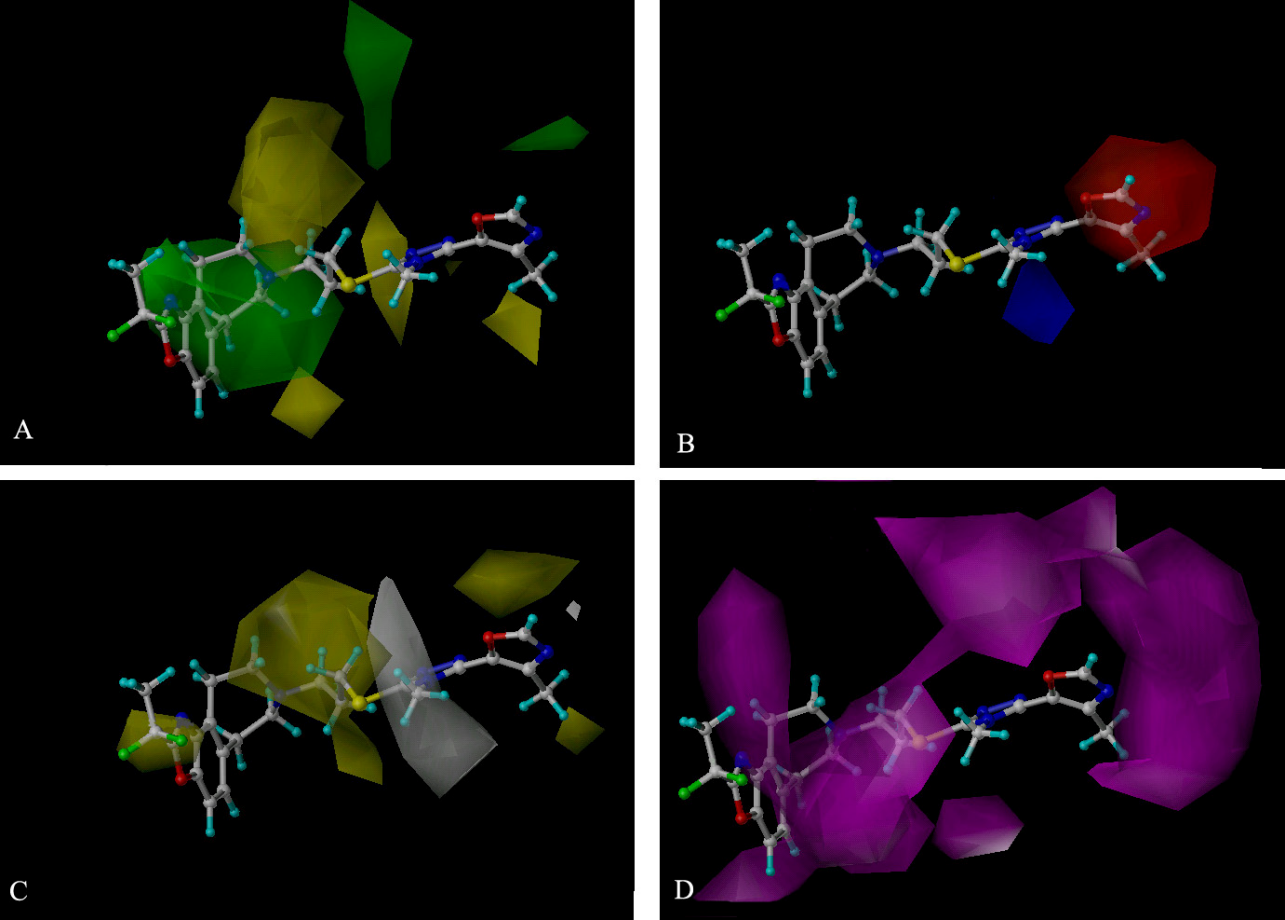
CoMSIA StDev*Coeff contour plots. (**A**) Steric (green/yellow) contour map in combination with compound 9. Green contours indicate regions where bulky groups increase activity; yellow contours indicate regions where bulky groups decrease activity; (**B**) Electrostatic contour map (red/blue) in combination with compound 9. Red contours indicate regions where negative charges increase activity; blue contours indicate regions where positive charges increase activity; (**C**) Hydrophobic contour map (yellow/white) in combination with compound 9. Yellow contours indicate regions where hydrophobic substituents enhance activity; white contours indicate regions where hydrophilic substituents enhance activity; (**D**) HB acceptor contour map (magenta/red) in combination with compound 9. Magenta contours indicate regions where HB receptors on the receptor promote the affinity; cyan contours indicate regions where HB acceptors on the receptor demote the affinity.

In our study, various stdev*coeff contour maps were constructed to view important features for the interaction between ligand and the target protein, based on the receptor-based optimal CoMSIA models. These contour maps allow identification of those positions that require a particular physicochemical property to enhance the bioactivity of a ligand [34] and, therefore, have been widely used in recent 3D-QSAR studies [15]. In our present work, the maps generated depicted regions with scaled coefficients: 85% (favored) or 15% (disfavored). As compound 9 (Figure 4) is one of the most active molecules in the whole dataset (fpKi value is 9.1), in all the following contour maps (Figure 5) it is shown as an example molecule to exploit the possible interactions between the benzazepine antagonists and dopamine D3 receptor.

### 2.4. CoMSIA Contour Maps Analysis

The contributions of the steric fields for the optimal CoMSIA model were graphically displayed in contour maps in Figure 5A, where the steric field defined by the green colored contours represents regions of favorable steric effect, while yellow colored contours represent regions of unfavorable steric effect, respectively. It can be easily found that a large positive steric (green) region appears above ring-A and ring-B, especially around the R_1_ substituent (position-2 in ring-A). Thus, molecules carrying a bulky ring or substituent at position-2 in ring-A should be more active than those with a smaller substituent, or without a substituent, like that molecules 18 (fpKi = 8.4) and 19 (fpKi = 7.6) with a bulky substituent such as -pir are more active than molecules 3 (fpKi = 7.2) and 4 (fpKi = 7.0) with a –CH_3_ in this position. To improve the inhibition potency of the set of compounds we therefore need to try to develop new analogs with increasing steric substituents in these regions. In contrast, two negative steric (yellow) regions appear mainly above the plane of ring-C, the position-15 and ring-D, drawing a conclusion that a substituent of bulk steric at this position disbenifits the biological activity of the molecules, which is illustrated by the fact that compounds 95 (fpKi = 6.6) and 96 (fpKi = 6.9) with a –CH_3_ in position-15 exhibit lower activities than molecules 78 (fpKi = 7.4), 79 (fpKi = 7.5), 85 (fpKi = 8.2) and 86 (fpKi = 7.6) which have no substituent in that position at all.

Electrostatic fields based on the PLS analysis of the CoMSIA models are shown in Figure 5B. A small red isopleth around the R_3_ substituent in ring-D indicates the area’s preference for negative charged substituents. For compounds 8 (fpKi = 8.8) and 11 (fpKi = 9.1), due to the strong electronegativity of nitrogen and oxygen atoms in R_3_ substituent, their activities are greater than compounds 7 (fpKi = 8.4) and 10 (fpKi = 9.0). A blue region appears below the plane of ring-D suggesting the preference for positive charges at this region.

The CoMSIA hydrophobic contour maps of affinity for DA D3 receptor are depicted in Figure 5C, where yellow and white contours highlight areas where hydrophobic and hydrophilic properties are preferred. Two yellow isopleths, above the R_1_ substituent in ring-A (position-2) and around the plane of ring-C and position-15, indicate that hydrophobic groups (like –OMe, –OEt, –F, –Cl, –Br) are beneficial here to enhance the activity. This is illustrated by the example of compound 15 with –CF_2_CH_3_ at this position showing a much higher activity than any other compound in the group of 1–19. Another yellow isopleth appears above the R_3_ substituent in ring-D indicating that hydrophobic groups in these positions are beneficial to enhance the activity. White polyhedra appear near ring-D, indicating that hydrophilic (like hydroxy or amido) groups here are correlated with good antagonist activity of the molecules.

Figure 5D depicts the HB acceptor contour maps of the CoMSIA models. Magenta contours encompass regions where a hydrogen bond acceptor will lead to improved biological activity, while a HB acceptor located near the cyan regions will result in impaired biological activity. From this figure, a large magenta contour is observed surrounding R_1_ substituent and ring-A, supporting the requirement of HB acceptor in the location to improve the activity. Compounds 7–16 which exhibit much higher activity than any others in the dataset are just such cases, due to their R_1_ substituent with F atom. Another large magenta contour surrounding the R_3_ substituent and ring-D indicates that HB acceptor is beneficial to the activity, which further illustrates why this new kind of DA D3 receptor antagonist with Part 4 basic structure (ring-D has three nitrogen atoms which seems to be a powerful HB receptor) may express higher activity.

**Figure 6.**
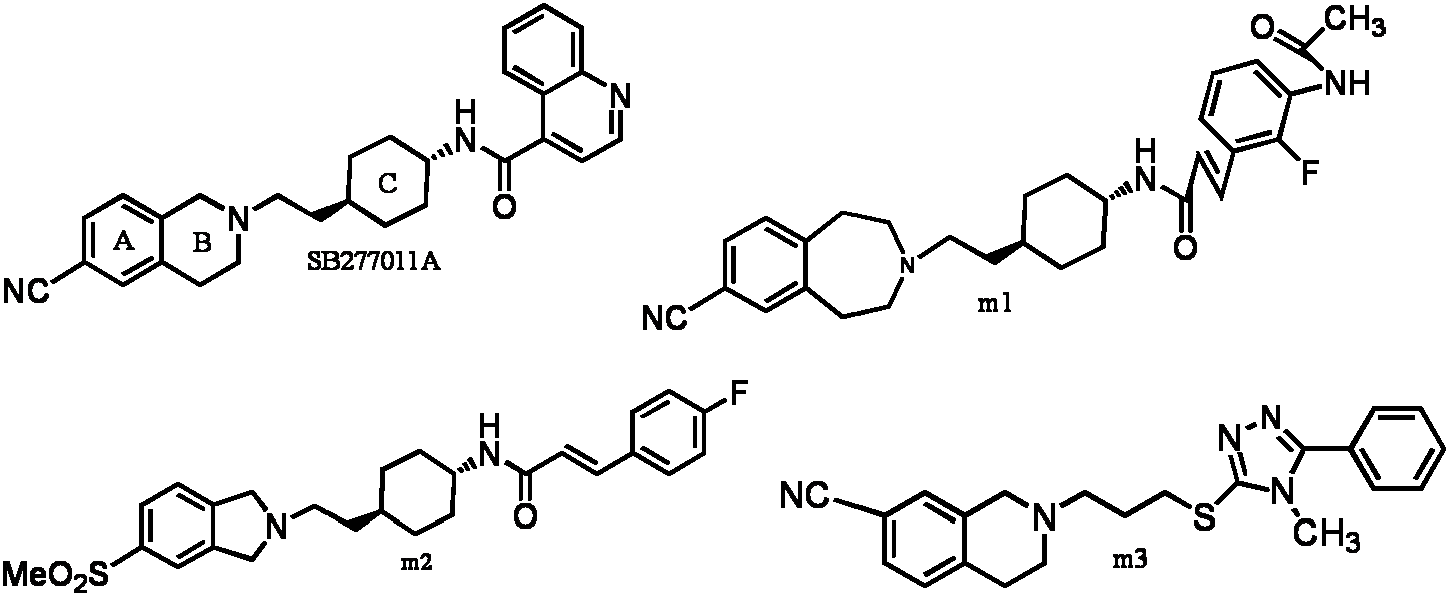
The structures of SB277011A, *m1*, *m2* and *m3* [1].

Recently, using the dopamine D3 receptor antagonist SB277011 as a starting point, and from examination of molecular models, two derivatives named by *m1* and *m2* (Figure 6) were synthesized and evaluated by Austin and his colleagues, which exhibited nanomolar affinities for D3 receptors (Ki = 4.0 nM for *m1* and Ki = 5.0 nM for *m2*, respectively) [35,36]. Integration of a triazole moiety into the N-alkyl spacer yielded selective D3 ligands as exemplified for *m3*, which also showed a greater D3 affinity of 4.3 nM [1]. In comparing *m1∼3* with SB277011 it is apparent that the substituent in arylcarbamide moiety may play an important role in improving the activity, and the variations of the ring size in ring-B exert an influence on the activity. Moreover, introduction of a trans-1,4-cyclohexylethyl spacer, as well as further optimizations of the arylcarbamide moiety and the position of attachment for the cyano- or methylsulfonyl-function, has yielded potent and selective D3 antagonists [1]. All the above conclusions confirm part of our results that ring-A plays a very important role in design of DA D3 antagonist with high activity. As to their conclusions that introduction of a trans-1,4-cyclohexylethyl spacer, position of attachment for the cyano- or methylsulfonyl-function will yield more potent and selective D3 antagonists, since the structures of the molecules they studied are quite different from our dataset both in skeleton and the substructures, we cannot draw a similar conclusion unless more data enclosing such specific compounds under the same experimental environment are supplemented into our models.

### 2.5. Homology Modeling Results

**Figure 7.**
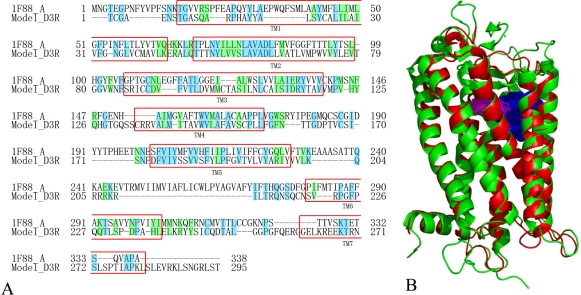
Homology modeling results. (**A**) Sequence alignment of bovine rhodopsin (1F88 chain A) and the DA D3 receptor homology model. The identical residues are shaded in blue, while similar residues are shaded in green. The seven TM domains (named TM1∼7) are boxed by a red border; (**B**) Superposition of template protein 1F88 chain A (green ribbon) and the DA D3 receptor model structure (red ribbon) from homology modeling. Blue and pink dot regions are the binding pocket of compound 9 and the template protein 1F88 chain A, respectively.

Figure 7 shows the structural superposition of the DA D3 receptor homology model to the X-ray crystal structure of template 1F88_A which was created by SuperPose web server 1.0 [37]. Seemingly, the sequence identity between DA D3 receptor homology model and the template 1F88_A is somewhat low, only 21.4%, with a similarity of 35.6%. However, after a careful analysis of the alignment of the model and template, the sequence identity in the important seven transmembrane (TM) domains which are regarded as the structurally conserved regions is found to be 33.1% with a similarity of 54.4%, satisfying the normal criterion that a sequence identity higher than 30% could be used to predict the protein structure [38]. Finally, we assessed the geometric qualities of the model using PROCHECK and the results showed that: for ramachandran plot: 86.6% core, 10.0% allow, 3.4% gener and 0.0% disall; for main-chain and side-chain params: 11 better, 0 inside and 0 worse. And as seen in Figure 7B, the template protein 1F88 chain A (green ribbon) are well superposed with the DA D3 receptor model structure (red ribbon) from homology modeling. All these results provided a good validation of the modeled structure of D3 dopamine receptor.

Figure 7A shows the important positions for the folding of the helices that were aligned. The length of the loop regions between the DA D3 receptor loop sequences and the template 1F88_A is comparable, except loop IL3 (the region between TM5 and TM6). This is because the DA D3 receptor IL3 loop (with 104 residues) is much longer than that of bovine rhodopsin (with only 12 residues) [39]. Here the *N*-terminus and the intracellular loop IL3 were omitted due to the fact that they are presumably remote from the binding site, and furthermore, were not found to be crucial for ligand binding in previous studies on chimeric receptors [39,40]. Varady *et al.* recently also demonstrated that omission of even more loops still allows for construction and simulation of a meaningful D3 model, which was used successfully by the authors for structure-based virtual screening [41]. Considering all these features, the homology model we built was found to be in good agreement with previous models [39,40]. In addition, many key amino acids (such as CYS101, ILE105, LEU106, VAL151, PHE175, PHE184, PRO254 and ALA251) interacting with the DA D3 receptor antagonists in the binding site, are well overlaid in 3D space in both structures.

### 2.6. Docking Results

In order to explore the real binding environment where the ligand interacts within the protein, docking studies were carried out on these compounds. Figure 7B shows the binding pocket we generated (blue dot region). It is observed that this binding pocket is partly overlapped with the binding pocket in the template protein 1F88 chain A. Furthermore, the binding site we generated in the seven TM domains corresponds well to the studies of Frank Boeckler *et al.* [1,39]. All these findings suggest that the binding site we found is appropriate for the study of DA D3 receptor antagonists. Presently, all 109 compounds were docked into the possible active site, and finally each compound got 20 possible active conformations with different total scores. The selection was made in such a way that docking conformations with comparatively higher scores were chosen for those compounds with higher antagonist activities, and *vice versa*. Finally, a correlation analysis between the docking scores and the fpKi values of the whole dataset was carried out, resulting in a *R*^2^ of 0.39 and a highest docking score of 4.4 for the antagonists. This result not only proves the reasonability of the docking, as well as the homology protein model, but also reveals a proper correlationship between the docking conformation and the antagonist potency of the fused benzazepine derivatives.

### 2.7. Molecular Dynamics Simulations

**Figure 8.**
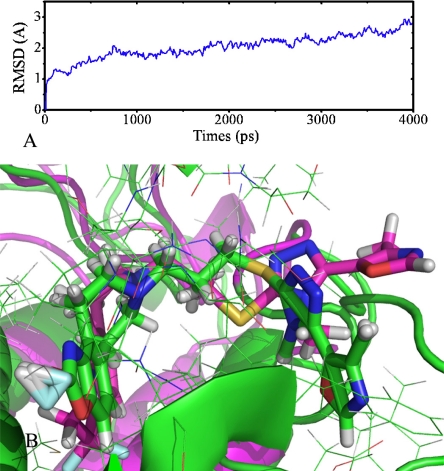
MD simulations results. (**A**) Plot of the RMSD of docked complex *versus* the MD simulation time in the MD-simulated structures; (**B**) View of superimposed backbone atoms of the average structure of the last 1000 ps of the MD simulation (green) and the initial structure (pink) for compound 9 and DA D3 receptor complex. Compound 9 is represented in pink for initial complex and green for the final average complex. Different colors represent different kinds of atoms: For compound 9, common C is shown in pink and for amino acid residues common C is shown in cyan, white for H, blue for N, red for O, cyan-blue for F and yellow for S, respectively.

In the present work, we performed 4 ns molecular dynamics simulations of DA D3 receptors with ligand 9 based on the docked complex structure to obtain a dynamical picture of the conformational changes. The main purpose of the simulations is to study the conformational alterations of ligand 9 in the DA D3 binding pocket. The RMSDs of the trajectory with respect to their initial structure ranging from 2.1 to 2.7 are depicted in Figure 8A. After 2.5 ns, the RMSD of the complex reached about 2.2 and almost retained this value for the entire simulation. This clearly indicates metastable conformation after 2.5 ns of simulation for docked complex structure. A superposition of the average structure of ensemble for the last 1 ns and the docked structure is shown in Figure 8B, where the pink ribbon represents initial structure for the docked complex, the green ribbon represents the MD-simulated structure, respectively. And compound 9 is represented in pink for initial complex and green for the final average complex, respectively.

In Figure 8B it can be observed that the docked complex and MD average structure are in the same binding site. There is no significant difference between the average structure and the docked complex. Ring-A, -B and -C are superimposed well in these two models. This indicates the reasonability of the homology model and the docking results. The only difference is that ring-D and R3 substituent in MD average structure is in front of the docked complex. In Figure 7A, the sequence similarity between TM5 and TM6 (IL3 part) is not very high. After the optimization in MD simulations this part becomes more reasonable and the space between TM5 and TM6 becomes smaller. In order to bind into this pocket, the ring-D and R3 substituent of compound 9 must bend to the TM5.

**Figure 9.**
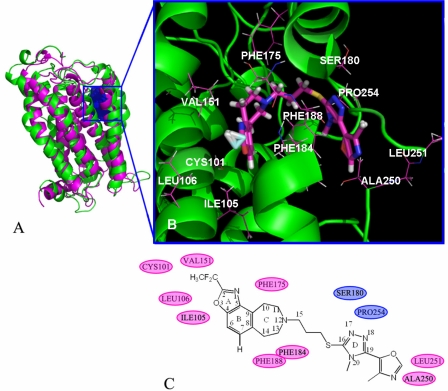
The binding pocket formed around molecule 9. (**A**) Superposition of the MD simulation (green) and the initial structure (pink) for DA D3 receptor. Blue dot regions are the binding pocket of compound 9; (**B**) Steric amino acid residues around the compound in the active docking pocket; (**C**) Positions of hydrophobic (pink border) and hydrophilic (blue border) amino acid residues.

As the most potent antagonist in the dataset, compound 9 is chosen as an illustration to analyze the MD results. Figure 9A shows the binding pocket in the protein. Figure 9B shows the steric amino acid residues around the compound in the binding pocket. It can easily be seen that no steric amino acid residues appear above ring-A, especially around the R_1_ substituent (position-2 in ring-A). However, several crucial amino acid residues are observed around some specific positions of the molecules. For example, PHE175, SER180, PHE184, PHE188 and PRO254 lie above ring-C, position-15 and ring-D. These results further confirm the results of the CoMSIA model (Figure 5A), where bulky substituents in position-2 (ring-A) improve the activity, but bulky groups at positions-11, -15 (ring-C) and ring-D impair the activity.

In Figure 9C, hydrophobic amino acid residues CYS101, ILE105, LEU106 and VAL151 appear above the R_1_ substituent in ring-A (position-2), and ALA250, LEU251 appear above the R_3_ substituent in ring-D, indicating that compounds with hydrophobic groups in these positions may have higher activity. Hydrophilic amino acid residues SER180 and PRO254 above ring-D suggest that compounds with hydrophobic groups in these regions may reduce the activity. These MD results correspond well to our previous CoMSIA analysis, where in Figure 5C (the hydrophobic field contour map), one yellow isopleth above the R_1_ substituent and ring-A (position-2) indicates the favor of the locations for hydrophobic groups, and white polyhedra appear above ring-D which implies a preference for hydrophilic groups in the areas.

These conclusions are well consistent with the findings obtained from the CoMSIA contour maps analysis, in that bulky R_1_ substituent in ring-A of antagonist can be able to interact with receptor simply due to the fact that it may well fit in the binding pocket. In addition R_1_ substituent and R_3_ substituent of antagonists with hydrophobic group are favorable to enhance the activity.

## 3. Materials and Methods

### 3.1. Compounds and Activity

After discarding six compounds with unspecified inhibitory activities, a total of 110 tricyclic derivatives synthesized as the DA D3 receptor antagonists [7,11] were used as dataset in the present work, with their fpKi values (functional pKi obtained from the GTP*γ*S functional assay) employed as the biological activity (Tables S1–S4, supplementary materials). Although all compounds in this dataset are also DA D2 antagonists, most of their fpKi values for DA D2 receptor are undefined due to experimental difficulty in the literatures, thus here we only study their D3 receptor antagonist properties. In structure, 110 tricyclic derivatives were divided into skeleton types A–K, where types A–D contain 26 compounds (Table S1), E∼J 72 compounds (Table S2) and K with only 4 molecules (Table S3) whose structures are independent of any other types in the whole dataset. In this work, only molecule 1 was omitted as it has no common substructure with others used. In a ratio of about 3:1, the remaining dataset, composed of 109 BAZ compounds, were divided into a training set (82 molecules) to build the subsequent QSAR model and a test set (27 molecules) for validating the predictability of the model. The compounds in the test set were chosen to make sure that their fpKi values are uniformly distributed in the whole biological activity range of the dataset. The fpKi values are considered as dependent variables in the CoMFA and CoMSIA analyses. Energy minimization was performed using SYBYL 6.9 package (Tripos Associates, St. Louis, MO, U.S.), tripos force field was used and conjugate gradient method with convergence criterion was set as 0.05 kcal/mol for this process. Partial atomic charges were calculated by the Gasteiger-Huckel method [21].

### 3.2. Homology Modeling

The accurate 3D structure of DA receptors is still unavailable [42], thus homology modeling, based on consensus alignment [43], was used here to build the D3 receptor structure. Homology modeling is an effective method for predicting a three dimensional structure, provided that homologous proteins exist whose 3D structures are known. In this work, the rhodopsin X-ray structure (PDB entry: 1F88, chain A, 2.8 Å) has been applied since it belongs to the same subfamily of the GPCRs with dopamine receptors [40,44]. The target DA D3 receptor which has 400 amino acids (Swiss-Prot Accession Number: P35462.2) was taken from the NCBI website (http://www.ncbi.nlm.nih.gov).

The template has about 32% sequence similarity with DA receptors for the whole sequence and about 55% for the structurally conserved regions, *i.e.*, the active center [39]. The modeling process was carried out by ESyPred3D web server 1.0 [45], which mainly used the MODELLER package [45], and also performed sequence alignment by combining, weighting and screening the results of several multiple alignment programs. Finally, the generated D3 dopamine receptor model was validated by PROCHECK program (http://nihserver.mbi.ucla.edu/SAVS/) [46]. The aim of PROCHECK is to assess how normal, or conversely, how unusual the geometry of the residues in a given protein structure is, as compared with stereo chemical parameters derived from well refined and high resolution structures [42]. All H-atoms were subsequently added to the unoccupied valence of heavy atoms at the corresponding neutral state using the biopolymer module of SYBYL 6.9 package.

### 3.3. Molecular Docking

To determine the probable binding conformations and offer more insight into understanding the interactions between DA D3 receptor and its antagonist, molecular docking analysis was carried out using the Surflex docking of SYBYL package. This docking approach aligns the ligand to a “protomol” or idealized ligand in the active site of the target. Our molecular docking executes the following steps: Firstly, the protein structure obtained from homology modeling was imported into Surflex and then hydrogens are added. Secondly, the protomol was generated using a ligand-based approach. During the protomol generating process, the specification of two parameters is critical for forming appropriate binding pocket. One is the protomol_bloat determining how far the site should extend from a potential ligand, and the other is the protomol_threshold determining how deep into the protein the atomic probes used to define the protomol can penetrate. The protomol_bloat value was set at 0 and the protomol_threshold value at 0.49 when a reasonable binding pocket was obtained. Finally, all the antagonists were docked into the binding pocket and each of them got 20 possible active docking conformations with different scores. During the docking process, the protein was considered as rigid and the antagonist molecules flexible, with all other parameters adopted default values.

### 3.4. Conformational Sampling and Alignment

**Figure 10.**
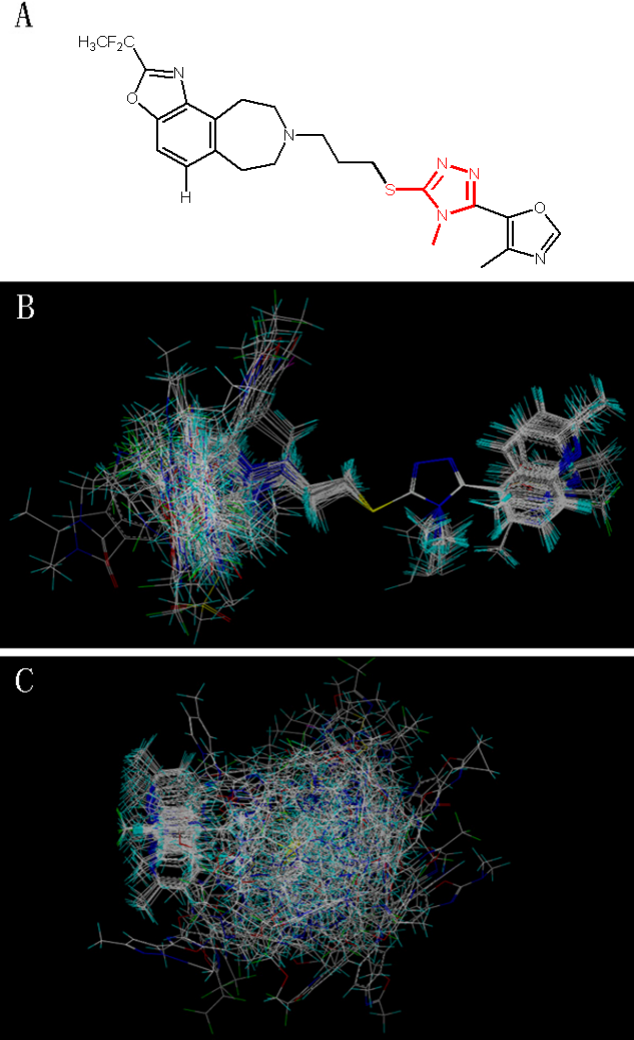
Molecular alignment of compounds in the whole dataset. (**A**) Common substructure of the molecules is shown in red based on template compound 9; (**B**) Ligand-based alignment of all the compounds; (**C**) Receptor-based alignment of all the compounds.

Based on an atom-by-atom superimposition principle, the alignment of the molecules was carried out by the ALIGN DATABASE command in SYBYL. In the present study, ligand-based alignment and receptor-based alignment rules were adopted. In the ligand-based alignment, molecule 9 with the highest fpKi values (fpKi = 9.1) was chosen as the template molecule. Figure 10A shows the common substructure depicted in red, and Figure 10B shows the resulting ligand-based alignment model.

The other alignment we used is the receptor-based alignment. After docking process, the conformations for all compounds with optimal scores in DA D3 receptor protein could not present a statistically significant result. Therefore, the optimal conformation of each molecule was selected from the 20 conformations in order to ensure the score and the activity have a good correlation. Finally we got a correlation coefficient R^2^ of 0.39. Then all molecules’ partial atomic charges were calculated by the Gasteiger-Huckel method [21]. The receptor-based alignment model is shown in Figure 10C.

### 3.5. Descriptor Calculation

CoMFA [47] and CoMSIA [48] were performed to build the models in order to reveal the relationship between 3D structural features and activities by employing the standard option of SYBYL. In CoMFA analysis, the superimposed molecules are kept in a 3D grid and steric and electrostatic fields are then calculated at various grid points using Lennard-Jonnes and Coulombic potentials, respectively [24]. CoMFA method only calculates steric and electrostatic interactions, yet CoMSIA also calculates hydrophobic, hydrogen-bond (HB) donor and HB acceptor interactions. The basic assumption of CoMSIA is that a suitable sampling of the steric, electrostatic, hydrophobic and HB acceptor interactions generated around a set of aligned molecules with a probe atom might provide all important features for understanding their biological activities, and that the changes in binding affinities of ligands are related to changes in molecular properties [49].

In our present work, the CoMFA and CoMSIA models were generated by SYBYL with default parameters. To derive the CoMFA and CoMSIA descriptor fields, a 3D cubic lattice with grid spacing of 2Å in x, y, and z directions, was generated automatically to encompass the aligned molecules. All CoMFA calculations were accomplished using a sp^3^ carbon atom with a charge of +1.00, a cut off value of 30 kcal/mol for the Lennard-Jones and Coulomb-type potential, and a constant dielectric function. The probe atom was placed at each lattice point, and their steric and electrostatic interactions with each atom in the molecule were computed using the CoMFA standard scaling. CoMSIA similarity indices descriptors were also derived within a lattice box with a grid spacing of 2 Å and a sp^3^ carbon with +1 charge as probe atom. A Gaussian function was used to evaluate the mutual distance between the probe atom and each molecule atom. CoMSIA similarity indices (AF) for a molecule j with atom i at a grid point q are calculated by Equation 1 as follows:

(1) $$A_{F,k}^{\text{q}}(j)=-\sum \omega_{\text{probe},k}\omega_{ik}e^{-\alpha r_{iq}^{2}}$$

where ω_probe,k_ is the probe atom with radius 1 Å, charge +1, hydrophobicity +1, hydrogen bond donating +1 and hydrogen bond accepting +1. ω_ik_ is the actual value of the physicochemical property *k* of atom *i. r*_iq_ is the mutual distance between the probe atom at grid point *q* and item *i* of the test molecule [50].

In addition, in order to deeply explore the impact of hydrophobic property of the molecules on their DA D3 antagonist potency, another hydrophobic parameter besides the hydrophobic field in CoMSIA analysis, *i.e.*, MlogP [31], was also calculated by Dragon professional version 5.4 [30], and used in the building of 3D QSAR models. Dragon is an application software for calculation of 1664 various molecular descriptors for each molecule. These descriptors can be used to evaluate molecular structure–activity or structure–property relationships, as well as for similarity analysis and high-throughput screening of molecule databases (Dragon user’s manual (http://www.talete.mi.it/help/dragon_help/index.html).

### 3.6. Calculation and Validation of 3D-QSAR Models

To obtain statistically significant 3D-QSAR models, the Partial Least Squares (PLS) regression was used to analyze the dataset by correlating the variation in their fpKi values (the dependent variable) with variations in their CoMFA/CoMSIA interaction fields (the independent variables). The advantage of this method is that it can reduce large numbers of original descriptors to a few principal components (PCs) that are linear combinations of the original descriptors [51]. The optimum number of PCs was determined by the leave-one-out (LOO) cross-validation procedure. Then with this optimal PC number, a non-cross-validation analysis was carried out, and the Pearson coefficient (*R*^2^_ncv_) and standard error of estimates (*SEE*) were calculated [51].

During PLS process, to evaluate the reliability of the model generated, several statistical parameters including the *Q*^2^ and above *R*^2^_ncv_ are crucial. As a cross-validated coefficient, *Q*^2^ is used as a statistical index of the predictive power of the model, and is calculated by Equation 2 where the *Y*_predicted_, *Y*_observed_ and *Y*_mean_ are predicted actual and mean values of the target property, respectively [49].

(2) $$q^{2}=1-\frac{\sum_{Y}{\left(Y_{\text{predicted}}-Y_{\text{observed}}\right)}^{2}}{\sum_{Y}{\left(Y_{\text{observed}}-Y_{\text{mean}}\right)}^{2}}$$

When assessing the predictive power of the QSAR model derived using the training set, an independent test set was used and their biological activities were predicted. The predictive *R*^2^ (*R*^2^_pre_) value is calculated using Equation 3.

(3) $$R_{\text{pre}}^{2}=(\text{SS}-\text{PRESS})/\text{SS}$$

where SS is the sum of squared deviations between the biological activity of the test set and the mean activity of training set molecules, and PRESS is the sum of squared deviations between the actual and the predicted activities of the test set molecules [52]. Finally, the CoMFA/CoMSIA results were graphically represented by field contour maps, where the coefficients were generated using the field type “Stdev*Coeff”.

### 3.7. Molecular Dynamics Simulations

Molecular dynamics simulations were carried out using Amber 10 [53] by starting with the docked structure of compound 9. Based on the general atom force field (GAFF) [54] and the AM1-BCC charge scheme [55] ligand parameters and charges were determined. The protein parameters were described by standard AMBER force field for bioorganic systems (ff03) [56]. The system neutralized with 10 counter chloridion ions, was solvated in a rectangular box of TIP3P water, keeping a minimum distance of 12 Å between the solute and each face of the box (92.33 × 82.63 × 86.55). The total number of the atoms of the simulation system was 53327 including the complex and waters. A cutoff distance of 10 angstrom was used to compute the nonbonded interactions, and periodic boundary conditions were applied. To remove possible bad contacts, the complex was minimized by a multistep procedure including 2000 steepest-descent steps followed by 2000 conjugate-gradient steps. Constant volume dynamics with a cutoff of 10Å was chosen. SHAKE was turned on for bonds involving hydrogen atoms [57].

Firstly, the minimized systems were gradually heated to 300 K at a constant force of 2.0 kcal mol^−1^ Ǻ^−2^. This was followed by a 50 ps pressure-constant period to raise the density while still constraining the complex atoms. After that, a 500 ps Langevin dynamics calculation with a collision frequency of 1 ps^−1^ was performed with a 2 fs time step in the NPT ensemble, at a constant temperature of 300 K. Finally, the production phase was taken 4 ns, with a 2 fs time step. Using default values, the long-range electrostatics was treated by the particle-mesh-Ewald method [58].

## 4. Conclusions

A 3D-QSAR study using CoMFA and CoMSIA methods was carried out for the first time on a series of 110 BAZ-based DA D3 receptor antagonists. Based on reasonable *Q*^2^, *R*^2^_ncv_, and *R*_pre_^2^ values, the obtained optimal models exhibited proper predictability. A good consistency was also found between our MD results and the 3D-QSAR models. By analysis of both the models and the derived contour maps, significant regions influencing the potency of dopamine D3 receptor antagonists were identified: (1) Ring-A, position-2 and R_3_ substituent in ring-D are key regions to the activity of the antagonists; (2) Molecules with large negative charge in R_3_ substituent or a bulky steric substituent at position-2 will lead to improved activity; (3) Hydrophobicity of the molecule represented by MlogP was found to be essential for building satisfactory QSAR models. And hydrophobic groups in both R_1_ substituent of ring-A and regions around ring-C plane and position-15 are especially sensitive areas for improving the binding affinity of the molecules. All these results can hopefully provide information for better understanding the interaction of D3 receptor-antagonists and help in the design of new DA D3 antagonists in the future.

## Figures and Tables

**Table 1.** Summary of CoMFA and CoMSIA results based on three different common substructures.

**PLS Statistics**	**A**		**B**		**C**	

	**CoMFA**	**CoMSIA**	**CoMFA**	**CoMSIA**	**CoMFA**	**CoMSIA**
*Q*^2^	0.371	0.404	0.364	0.373	**0.506**	**0.511**
*R*^2^_ncv_	0.805	0.780	0.815	0.780	**0.838**	**0.819**
*SEE*	0.346	0.366	0.338	0.366	**0.316**	**0.331**
*F*	43.739	44.388	46.437	44.427	**54.837**	**56.746**
*R*^2^_pre_	0.779	0.790	0.725	0.690	**0.794**	**0.715**
*SEP*	0.305	0.318	0.336	0.361	**0.296**	**0.367**
*OPN*	7	6	7	6	**7**	**6**
Contribution:						
Steric	0.435	0.115	0.434	0.110	**0.456**	**0.110**
Electrostatic	0.501	0.337	0.502	0.335	**0.481**	**0.341**
Hydrophobic		0.240		0.238		**0.233**
HB acceptor		0.236		0.244		**0.242**
MlogP	0.064	0.072	0.064	0.072	**0.064**	**0.072**

*Q*^2^, cross-validated correlation coefficient after the leave-one-out procedure; *R*^2^_ncv_, non-cross-validated correlation coefficient; *SEE*, standard error of estimate; *F*, ratio of *R*^2^_ncv_ explained to unexplained = *R*^2^_ncv_/(1 − *R*^2^_ncv_); *R*^2^_pre_, predicted correlation coefficient for the test set of compounds; *SEP*, standard error of prediction; *OPN*, optimal number of principal components.

**Table 2.** Summary of CoMFA and CoMSIA results based on three different charges.

**PLS Statistics**	**A**		**B**		**C**		**D**	

	**CoMFA**	**CoMSIA**	**CoMFA**	**CoMSIA**	**CoMFA**	**CoMSIA**	**CoMFA**	**CoMSIA**
*Q*^2^	**0.506**	**0.511**	0.485	0.414	0.437	0.395	0.417	0.475
*R*^2^_ncv_	**0.838**	**0.819**	0.772	0.695	0.783	0.833	0.827	0.930
*SEE*	**0.316**	**0.331**	0.373	0.425	0.363	0.321	0.327	0.212
*F*	**54.837**	**56.746**	42.301	43.834	45.149	52.739	50.525	94.645
*R*^2^_pre_	**0.794**	**0.715**	0.712	0.554	0.735	0.539	0.791	0.465
*SEP*	**0.296**	**0.367**	0.339	0.451	0.330	0.459	0.295	0.527
*OPN*	**7**	**6**	6	4	6	7	7	10
Contribution:								
Steric	**0.456**	**0.110**	0.450	0.106	0.445	0.100	0.449	0.112
Electrostatic	**0.481**	**0.341**	0.485	0.351	0.493	0.312	0.493	0.335
Hydrophobic		**0.233**		0.228		0.247		0.256
HB acceptor		**0.242**		0.226		0.287		0.249
MlogP	**0.064**	**0.072**	0.065	0.089	0.063	0.054	0.058	0.047

*Q*^2^, cross-validated correlation coefficient after the leave-one-out procedure; *R*^2^_ncv_, non-cross-validated correlation coefficient; *SEE*, standard error of estimate; *F*, ratio of *R*^2^_ncv_ explained to unexplained = *R*^2^_ncv_/(1 − *R*^2^_ncv_); *R*^2^_pre_, predicted correlation coefficient for the test set of compounds; *SEP*, standard error of prediction; *OPN*, optimal number of principal components.

**Table 3.** Summary of CoMFA and CoMSIA results.

**PLS Statistics**	**Ligand-based Model**		**Receptor-based Model**	

	**CoMFA**	**CoMSIA**	**CoMFA**	**CoMSIA**
*Q*^2^	0.506	0.511	0.418	0.603
*Q*^2^_cv(10)_	0.493	0.343	0.388	0.599
*R*^2^_ncv_	0.838	0.819	0.856	0.829
*R*^2^_boot_	0.892	0.872	0.899	0.882
*SEE*	0.316	0.331	0.292	0.316
*F*	54.837	56.746	114.875	125.886
*R*^2^_pre_	0.794	0.715	0.481	0.690
*SEP*	0.296	0.367	0.548	0.406
*OPN*	7	6	4	3
*Q*^2^_scrambling_	−0.526	−0.498	0.471	0.521
*CSDEP*	0.972	0.957	0.559	0.528
d*q*^2^/d*r*^2^*_yy_*	−0.145	0.046	1.048	0.678
Contribution:				
Steric	0.456	0.110	0.433	0.121
Electrostatic	0.481	0.341	0.546	0.383
Hydrophobic		0.233		0.181
HB acceptor		0.242		0.294
MlogP	0.064	0.072	0.022	0.022

*Q*^2^_cv(10)_, cross-validated correlation coefficient after the leave-group-out procedure (group number is 10); *R*^2^_boot_, *R*^2^ of boot strapping analysis (100 runs); *Q*^2^_scrambling,_ equals to (1 − (s*SDEP*)^2^). Predictivity of the model using the scaled standard deviation of error of prediction (s*SDEP*) instead of the *SDEP*; *CSDEP*, calculated cross-validated standard deviation of error of prediction; d*q*^2^/d*r*^2^_yy_, slope of *q*^2^ (calculated by SAMPLS using perturbed *y*-values, therefore denoted *q*^2^) versus the correlation of the perturbed to the original *y*-variables (denoted *r*^2^_yy_). *Q*^2^, cross-validated correlation coefficient after the leave-one-out procedure; *R*^2^_ncv_, non-cross-validated correlation coefficient; *SEE*, standard error of estimate; *F*, ratio of *R*^2^_ncv_ explained to unexplained = *R*^2^_ncv_/(1 − *R*^2^_ncv_); *R*^2^_pre_, predicted correlation coefficient for the test set of compounds; *SEP*, standard error of prediction; *OPN*, optimal number of principal components.

**Table 4.** Statistical results of CoMFA and CoMSIA models without MlogP used.

**PLS Statistics**	**Ligand-based Model**		**Receptor-based Model**	

	**CoMFA**	**CoMSIA**	**CoMFA**	**CoMSIA**
*Q*^2^	0.351	0.443	0.366	0.576
*R*^2^_ncv_	0.792	0.838	0.923	0.892
*SEE*	0.356	0.314	0.213	0.251
*F*	47.659	64.772	231.880	215.375
*R*^2^_pre_	0.668	0.471	0.416	0.604
*SEP*	0.384	0.537	0.606	0.475
*OPN*	6	6	4	3
Contribution:				
Steric	0.510	0.122	0.486	0.140
Electrostatic	0.490	0.318	0.514	0.367
Hydrophobic		0.288		0.205
HB acceptor		0.272		0.287

*Q*^2^, cross-validated correlation coefficient after the leave-one-out procedure; *R*^2^_ncv_, non-cross-validated correlation coefficient; *SEE*, standard error of estimate; *F*, ratio of *R*^2^_ncv_ explained to unexplained = *R*^2^_ncv_/(1 − *R*^2^_ncv_); *R*^2^_pre_, predicted correlation coefficient for the test set of compounds; *SEP*, standard error of prediction; *OPN*, optimal number of principal components.
